# The promotion of sestrin2/AMPK signaling by HIF-1α overexpression enhances the damage caused by acute myocardial infarction

**DOI:** 10.1186/s12872-023-03604-1

**Published:** 2023-11-20

**Authors:** Jie Wang, Honglei Du, Qing Sun, Weiping Wan, Haifeng Zhang

**Affiliations:** 1 Cardiac Intensive Care Unit, Yantaishan Hospital, Yantai, Shandong China; 2 Department of Cardiology, Yantai Yeda Hospital, No.23-1, the Yellow River Road, Yantai economic and Technological Development Zone, Yantai, Shandong 264006 China; 3 Department of Cardiology, Yantaishan Hospital, Yantai, China; 4 Department of Ultrasound, Yantaishan Hospital, Yantai, Shandong China

**Keywords:** Myocardial infarction area, Cell apoptosis, Cardiac function parameters, Myocardial tissue fibrosis

## Abstract

**Objective:**

Acute myocardial infarction (AMI), is a serious form of coronary heart disease. The present study sought to investigate the impact of HIF-1α on AMI, along with its fundamental mechanism.

**Methods:**

Sprague-Dawley (SD) rats were used to conduct an AMI model. 2,3,5-triphenyl-2H-tetrazolium chloride (TTC) staining was used examine the region of myocardial infract area at various time intervals. Protein expression levels were detected using western blotting. The rats were randomly divided into sham, model, negative control (NC), HIF-1α overexpression (HIF-1α-OE), and HIF-1α-OE+ si-sestrin2 groups. We examined the impact of HIF-1α overexpression on AMI rats using Haematoxylin-Eosin (H&E) staining, TTC staining, enzyme-linked immunosorbent assay (ELISA), TdT-mediated dUTP Nick-End Labeling (TUNEL) assay, and immunohistochemistry (IHC) staining.

**Results:**

According to the TTC findings, the region affected by myocardial infarction reached its peak at day 14. Based on the results from the western blot analysis, the levels of HIF-1α and sestrin2 were found the minimum on day 28. Subsequently, we discovered that the overexpression of HIF-1α rescued the cardiac function parameters, improved the morphology of myocardial tissue, and mitigated inflammation. Furthermore, the overexpression of HIF-1α led to a reduction in the levels of MDA and an increase in the levels of SOD. Moreover, the overexpression of HIF-1α resulted in a decrease in cellular apoptosis. This result was confirmed by the expression levels of Bcl-2 and Bax. Nevertheless, the defensive impact of elevated HIF-1α expression was somewhat counteracted by the suppression of sestrin2. In terms of mechanism, the overexpression of HIF-1α enhanced the levels of sestrin2 and the protein adenosine monophosphate activated kinase (AMPK).

**Conclusion:**

Our research suggests that the overexpression of HIF-1α may rescue the damage to myocardial tissue, and this effect is associated with the sestrin2/AMPK signaling pathway. Our study provides a novel comprehension of the protective effects of HIF-1α overexpression on AMI.

## Introduction

Acute myocardial infarction (AMI) is a prevalent ailment of the cardiovascular system [1]. Coronary artery stenosis-induced myocardial ischemic necrosis is a significant contributor to heart failure [2]. At present, there is a lack of specific pharmaceutical treatments available for addressing myocardial tissue injury after myocardial infarction [3]. Myocardial infarction possesses the capability to trigger oxidative stress and an inflammatory reaction within the myocardium, resulting in subsequent injury and programmed cell death of cardiac cells [4, 5].

Hypoxia Inducible Factor-1 (HIF-1), plays a vital role in maintaining oxygen equilibrium by controlling the transcriptional activity of various target genes, enabling cells to adapt to ischemia and/or hypoxia [6]. Numerous research studies have shown that HIF-1α can provide cardiac protection by reducing oxidative stress, inflammatory reaction, and cellular apoptosis caused by ischemia/reperfusion (I/R) [7, 8]. After myocardial ischemia, the activation of HIF-1α initiates the innate protective response of the body. This mechanism can regulate the body’s inflammatory signal pathway, thereby inhibiting the release of inflammatory factors. Previous studies have shown that HIF-1α overexpressed exosomes can effectively restore impaired angiogenesis, migration, and proliferation of hypoxic preconditioning HUVEC in vitro. Furthermore, these exosomes play a role in cardiac protection by increasing the expression of angiogenic factors and facilitating new angiogenesis [8].

Sestrin2, a member of the sestrin family, is a protein that is upregulated in various stress conditions including DNA damage, oxidative stress, and hypoxia [9]. According to prior research, sestrin2 regulates various biological and pathological processes through the signaling pathway of AMP-activated protein kinase (AMPK)/mTOR [10, 11]. In particular, sestrin2 stimulates receptors via AMPK/Peroxisome proliferators γ coactivator 1- α (PGC-1 α), which increases the resistance of the aging heart to ischemia and amplifies its vulnerability to ischemic damage [12].

Previous studies have shown the notable participation of HIF-1a in the anti-inflammatory reaction of macrophages and the survival of cells triggered by sestrins2 [13]. Wang et al. revealed that the increased presence of HIF-1 α in extracellular vesicles derived from mesenchymal stem cells enhances the efficacy of myocardial repair after myocardial infraction by utilizing microRNA [14]. The precise involvement of sestrin2/AMPK signaling in AMI with regards to HIF-1 α remains uncertain. The present study successfully established a rat model of AMI through ligation of the left anterior descending branch of the coronary artery, and subsequently observed an upregulation of HIF-1α. The primary objective of this investigation was to examine the potential impact of Sestrin2/AMPK on various aspects of cardiac function, histology, inflammatory factors, and cardiomyocyte apoptosis in the aforementioned rat model.

## Material and methods

### Animals

Sprague-Dawley (SD) rats were obtained from Jinan Peng Yue Experimental Animal Breeding Co. Ltd. The animals (SCXK (Lu) - 20,180,030) were housed in chambers with a temperature of 23 °C and a 12 h light/ 12 h dark cycle. They had unrestricted access to water and were given a standard diet for rodents. The Ethics Committee of Yantaishan Hospital (YSLZ2023152) approved all the procedures involving animals, and the animal experiments were conducted according to the ARRIVE guidelines (https://arriveguidelines.org).

### Generation and transfection of recombinant lentivirus

HIF-1αcomplete cDNAs were cloned and recombined with a lentivirus expression vector acquired from GeneChem in Shanghai, China. An empty lentivirus was employed as a control. Lentiviruses acquired from GeneChem (Shanghai, China) were employed to encode and combine Sestrin2 siRNA and its non-targeting siRNA.

### Animal model and grouping

The AMI model was created by ligating the left anterior descending coronary artery [15]. Following anesthesia administration (1%, 40 mg/kg pentobarbital sodium with intraperitoneal injection) in rats, a neck incision was performed to establish a connection with the ventilator. Subsequently, thoracotomy was conducted to expose the heart, and the left anterior descending branch of the coronary artery was ligated. Successful construction of the AMI model was indicated by the elevation of the ST segment in the electrocardiogram, deceleration of the heart rate, and whitening of the left ventricle. Left Ventricular End-Diastolic Diameter (LVEDD), left ventricular ejection fraction (LVEF), LV end-systolic Diameter (LVESD), LV fractional shortening (LVFS) [16]. The successfully modeled rats were randomly allocated into four groups (*n* = 6), namely the model group, NC group, HIF-1α overexpression (HIF-1α-OE) group, HIF-1α overexpression + si-sestrin2 (HIF-1α-OE + si-sestrin2) groups, each consisting of nine animals. Additionally, a sham surgery group comprising nine SD rats that underwent thoracotomy without ligation of the left anterior descending coronary artery. Prior to surgery, a negative control, a recombinant plasmid overexpressing HIF-1 α, and si-sestrin2 lentivirus were administered via the tail vein to the NC group, HIF-1α-OE group, and HIF-1α-OE + si-sestrin2 group, respectively. The model group and sham group received an equivalent volume of normal saline. Subsequently, injections were administered weekly, and rats were euthanized 28 days post-surgery to obtain myocardial tissue, which was stored at − 80 °C for future use.

### 2,3,5-triphenyl-2H-tetrazolium chloride (TTC)

After AMI, the heart was immediately removed and frozen at − 80 °C for later evaluation of the myocardial infarction’s severity using TTC (Solarbio, Beijing, China) staining. In order to achieve consistency, every heart was divided into five slices of the same thickness. These slices were subsequently immersed in a 1% TTC solution and incubated at a temperature of 37 °C for a duration of 25 minutes. After being exposed to 10% formaldehyde for a duration of 24 hours, the slices were organized based on their decreasing size and captured using an Olympus camera. Image J software was utilized to determine the region affected by infarction. Myocardial infarction area (%) = myocardial infarction area/total myocardial area × 100% [17].

### Haematoxylin-eosin (H&E) staining

Myocardial tissue of rats was fixed in a 4% paraformaldehyde solution and subsequently embedded in paraffin slices with a thickness of 4 μm. Then, H&E staining (Solarbio, Beijing, China) procedure was carried out. A light microscope (Leica, Germany) was used to observe myocardial tissue damage and inflammatory infiltration.

### Detection of LDH, CK-MB, TNF-α, IL-6, SOD, and MDA

After 24 hours of effective modeling, blood was collected from the abdominal aorta and left to sit at room temperature for a period of 2 hours. Afterward, the blood underwent centrifugation at 2500×*g* for 15 minutes to collect serum. The serum levels of LDH (BC0680, Solarbio, Beijing, China), CK-MB (SEKR-0059, Solarbio, Beijing, China), TNF-α (PT516, Beyotime, Shanghai, China), IL-6 (PI328, Beyotime, Shanghai, China), SOD (A001–3-2, Nanjing Jiancheng Bioengineering Institute, Nanjing, China), and MDA (A003–1-2, Nanjing Jiancheng Bioengineering Institute, Nanjing, China) were assessed using corresponding kits, following the manufacturers’ instructions.

### TdT-catalyzed dUTP Nick-end labeling (TUNEL) assay

Myocardial tissue section of rats underwent dewaxing and repair, then TdT enzyme (Beyotime, Shanghai, China) was added for a 60-minute reaction at 37 °C. Afterward, streptavidin labeled with horseradish peroxidase (HRP) was introduced and left to interact for 30 minutes at 37 °C. After 3,3′ Diaminobenzidine (DAB) staining (Beyotime, Shanghai, China) and hematoxylin staining for a duration of 10 minutes, the cells undergoing apoptosis displayed brown particles.

### Immunohistochemistry (IHC) staining

After dewaxing and repairing the antigen, the paraffin tissue section was diluted with a solution of 1:1000. Then, the rabbit anti- α-SMA (ab7817, Abcam, UK), HIF-1α (ab16897, Abcam, UK), and sestrin2 (ab178518, Abcam, UK) antibody diluent was added. The resulting mixture was incubated at 4 °C overnight, after which a second antibody (1:1500 dilution) was added for further incubation. The resulting mixture was subjected to DAB coloration, hematoxylin re-staining, and microscopic observation of a-SMA, HIF-1α, and Sestrin2. Positive cells were identified by their brown-yellow coloration with a microscope (Leica, Germany).

### Western blot

Total protein from myocardial tissue was lysed by RIPA lysis buffer from Beyotime (Shanghai, China). The protein content was assessed using the BCA kit (Solarbio, Beijing, China), the sample was subsequently combined with 2 × SDS loading buffer. The membrane transfer was conducted using SDS polyacrylamide gel electrophoresis. Subsequently, the PVDF membrane (Millipore, USA) was detached to indicate its orientation and then sealed with 5% TBST skimmed milk powder. The primary antibodies HIF-1α (ab16897, Abcam, UK), sestrin2 (ab178518, Abcam, UK), p-AMPK (Cell Signaling Technology, USA), and AMPK (Cell Signaling Technology, USA) were incubated overnight at 4 °C. The membranes were washed with TBST, followed by the addition of the second antibody, which was labeled with HRP and incubated at 37 °C for 60 minutes, then washed the membrane with TBST. The color development process was facilitated by the utilization of the enhanced chemiluminescence solution (ECL, Solarbio, Beijing, China), and the resulting image was captured. The densitometry analysis of the western blots was performed using the Image J software (NIH, USA).

### Statistical analyses

The statistical analysis of the data was conducted using SPSS 23.0 software (IBM, USA), and the drawing was completed with GraphPad Prism 8 software. The mean ± SD was used to present the experimental results. Statistical significance was determined using a one-way ANOVA followed by Turkey’s post hoc test for comparing multiple groups for data followed a normal distribution, with a significance level of *P* < 0.05.

## Results

### Changes in myocardial infarction area at different times

**Fig. 1:**
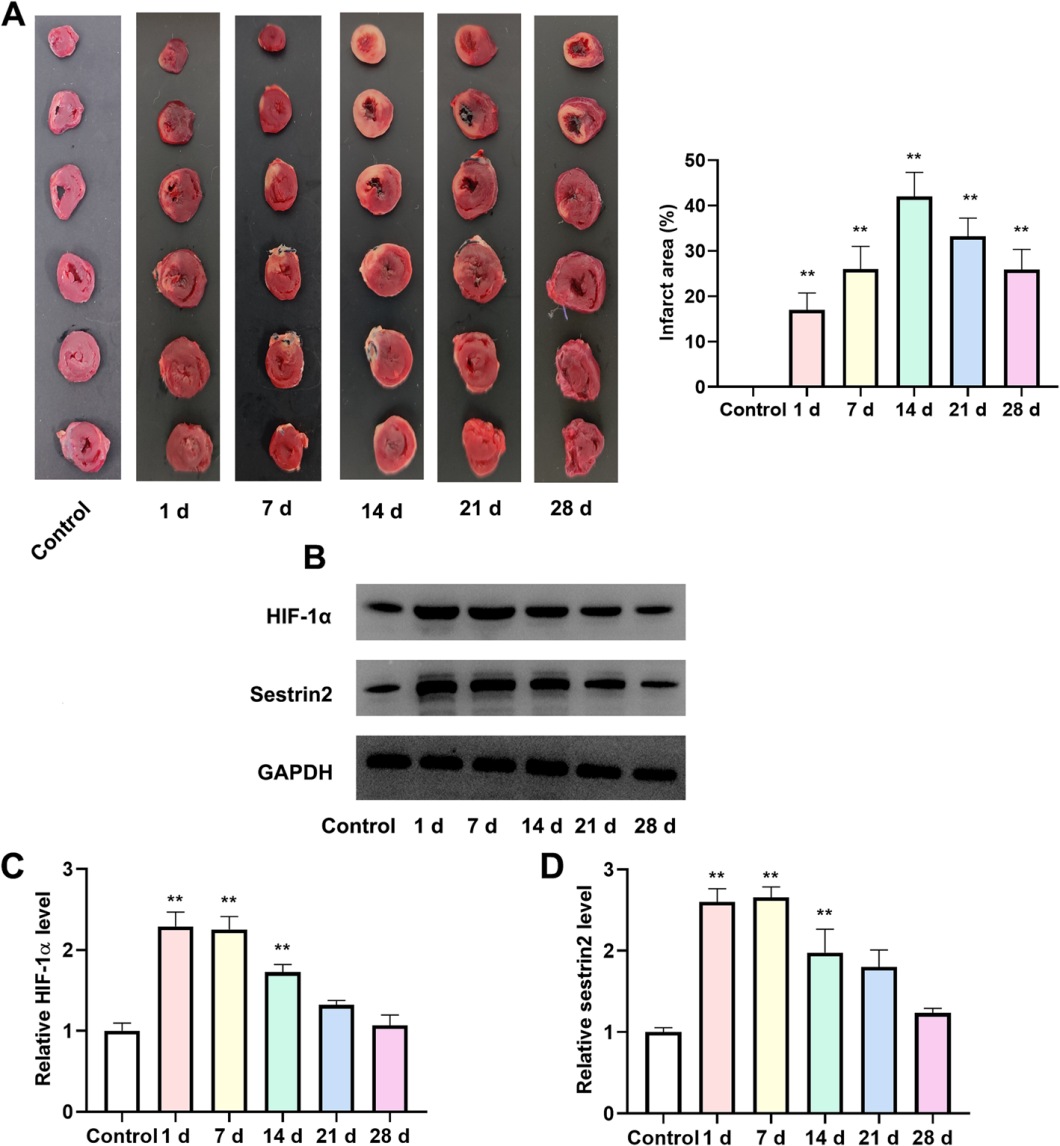
Changes of myocardial infarction area and protein expression levels at different times. **A** TTC staining was used to assess the myocardial infarction (*n* = 6); **B** Western blotting was utilized to detect the expression levels of HIF-α and sestrin2 at different time points (*n* = 3). The column diagram shows the quantification of HIF-α (**C**) and sestrin2 (**D**). Compared to the control group, ^**^P < 0.01

After TTC staining, the infarcted area was gray white, and the non-infarcted area was red. Except for the control group, the left ventricular anterior wall of rats at different times were showed different degrees of infarcted area after AMI. The results from Fig. 1A showed that myocardial infarction area was the largest at 14 d (*P* < 0.01). The area of myocardial infarction at day 21 and day 28 were decreased.

### Variations in the levels of HIF-1α and sestrin2 at various time points

To examine the impact of HIF-1α and sestrin2 on AMI, we measured the levels of HIF-1α and sestrin2 at various time intervals. According to the data shown in Fig. 1B-D, the levels of HIF-1α and sestrin2 increased following AMI in comparison to the control group (*P* < 0.01). There was no significant alteration in the levels of HIF-1α and sestrin2 on day 7 when compared to day 1. From day 14, there was a decrease in the levels of HIF-1α and sestrin2, and by day 28, their levels reached the lowest point. Nevertheless, there was no significant disparity in the levels of HIF-1α and sestrin2 when compared to the control group.

### Cardiac function parameters were improved by HIF-1αoverexpression

**Fig. 2:**
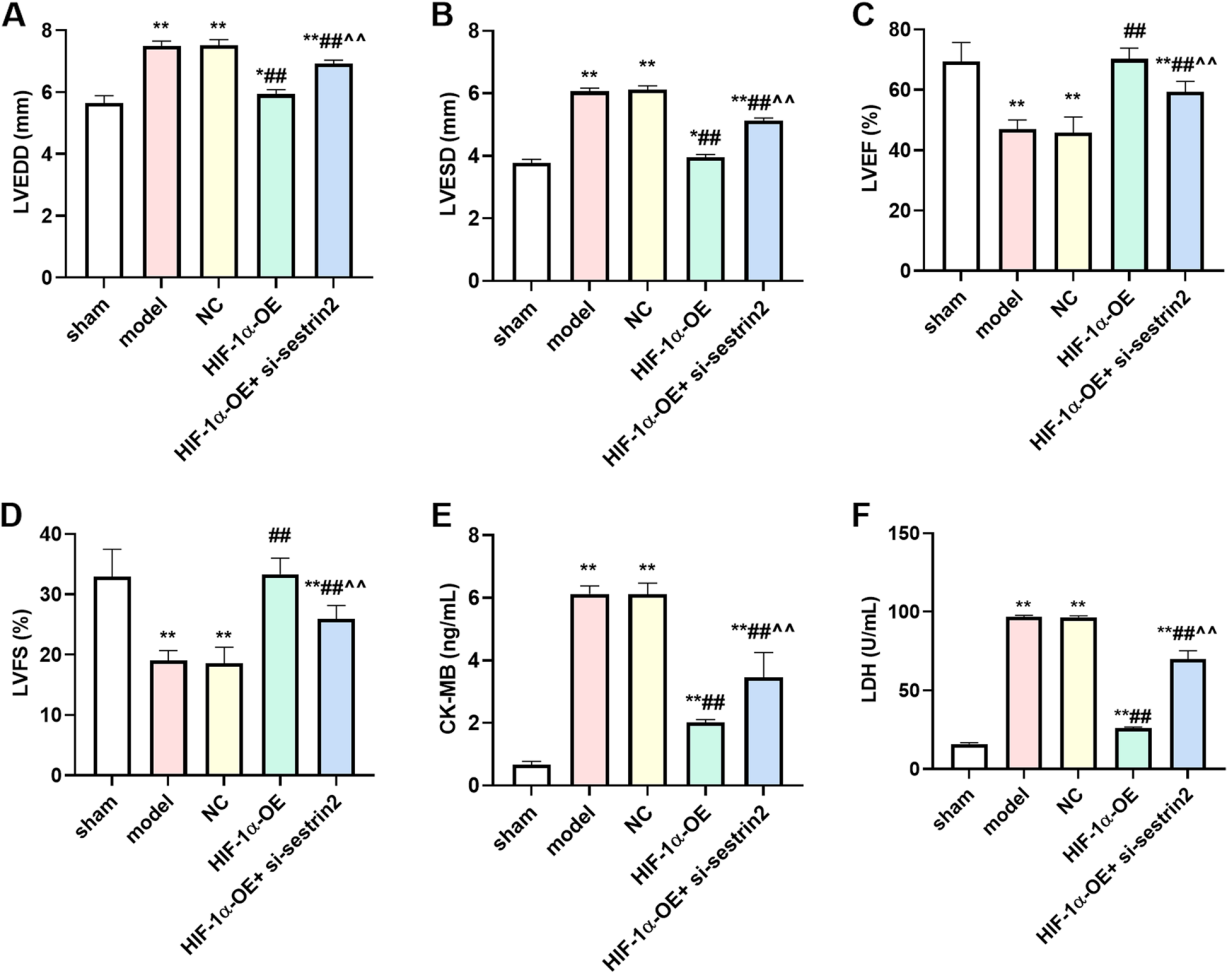
HIF-1α overexpression improved cardiac function parameters. The changes of LVEDD (**A**), LVESD (**B**), LVEF (**C**), and LVFS (**D**); **F** The content of CK-MB was assessed by corresponding CK-MB kit; **G** The content of LDH was assessed by corresponding LDH kit. *n* = 6. Compared to the control group, ^*^P < 0.05, ^**^P < 0.01; Compared to model or NC group, ^##^*P* < 0.01; Compared to HIF-1α-OE group, ^^^^*P* < 0.01

The cardiac function indicators LVEDD and LVESD showed a significant increase in both the model and NC groups, whereas LVEF and LVFS exhibited an increase compared to the sham group (*P* < 0.01). In the HIF-1α-OE group, there was a clear reduction in LVEDD and LVESD, accompanied by an increase in LVEF and LVFS, when compared to both the model and NC groups (*P* < 0.01). In contrast, the HIF-1α-OE + si-sestrin2 group exhibited a notable rise in LVEDD and LVESD, accompanied by a decrease in LVEF and LVFS, in comparison to the HIF-1α-OE group (Fig. 2A-D, *P* < 0.01). The serum LDH and CK-MB activities are commonly used to reflect the degree of myocardial injury. The model group exhibited a significant increase in serum LDH and CK-MB activities compared to the sham group (*P* < 0.01). Following the treatment of HIF-1α overexpression, there was a significant decrease in serum LDH and CK-MB activities compared to the model group (*P* < 0.01). Nonetheless, administration of si-sestrin2 partially counteracted the effects of HIF-1α overexpression (Fig. 2E, F).

### HIF-1α overexpression improved the morphological alterations in myocardial tissue and reduced inflammation in rats with AMI

**Fig. 3:**
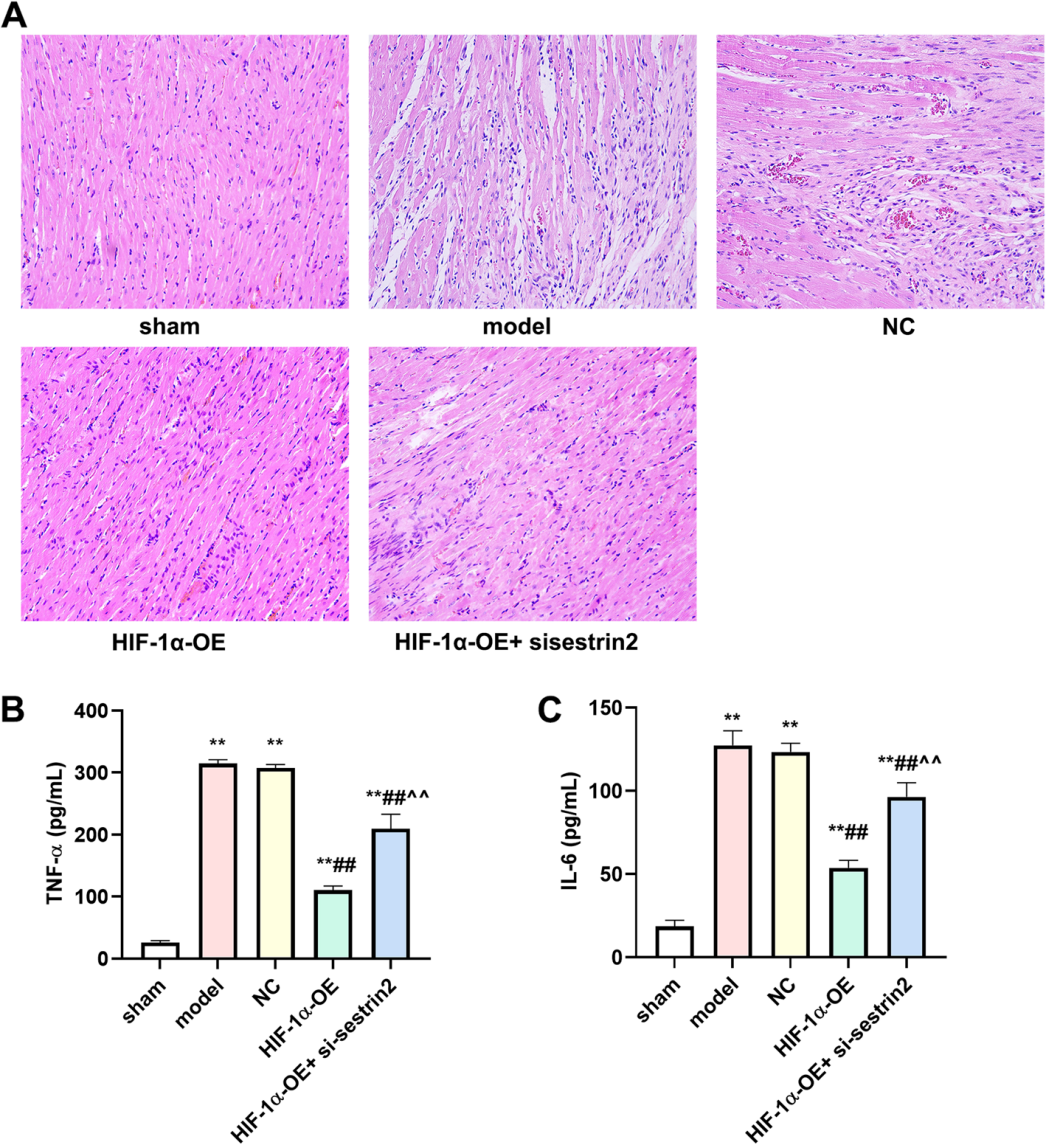
HIF-1α overexpression improved morphological changes of myocardial tissue and inflammation in AMI rats. **A** H&E staining was used to observe morphological changes of myocardial tissue; **B** The content of TNF-α was assessed by corresponding TNF-α kit; **C** The content of IL-6 was assessed by corresponding IL-6 kit. *n* = 6. Compared to the control group, ^**^P < 0.01; Compared to model or NC group, ^##^*P* < 0.01; Compared to HIF-1α-OE group, ^^^^*P* < 0.01

The sham group exhibited normal myocardial tissue morphology, with myocardial cells arranged in a regular and orderly manner, and a small gap between cells. In the model group, the myocardial cells exhibited swelling, necrosis, nucleation, extensive rupture of myocardial fibers, disorganized arrangement of myocardial fibers, and a notable infiltration of inflammatory cells. Following the pre-treatment of HIF-1α overexpression, there was an enhancement in the condition of myocardial cells and a decrease in myocardial cell necrosis. Nevertheless, the administration of si-sestrin2 diminished the beneficial impact of HIF-1α overexpression (Fig. 3A). Additionally, we identified the levels of TNF-α and IL-6 in the serum. The levels of TNF-α and IL-6 in the model and NC groups were significantly higher than those in the sham group, as demonstrated in Fig. 3B and C (*P* < 0.01). As anticipated, the levels of TNF-α and IL-6 significantly decreased in the HIF-1α-OE group when compared to both the model and NC groups (*P* < 0.01). In addition, the levels of TNF-α and IL-6 were significantly increased in the HIF-1α-OE + si-sestrin2 group compared to the HIF-1α-OE group (*P* < 0.01).

### HIF-1α overexpression attenuated the size of myocardial infarction region and ameliorated the state of oxidative stress

**Fig. 4:**
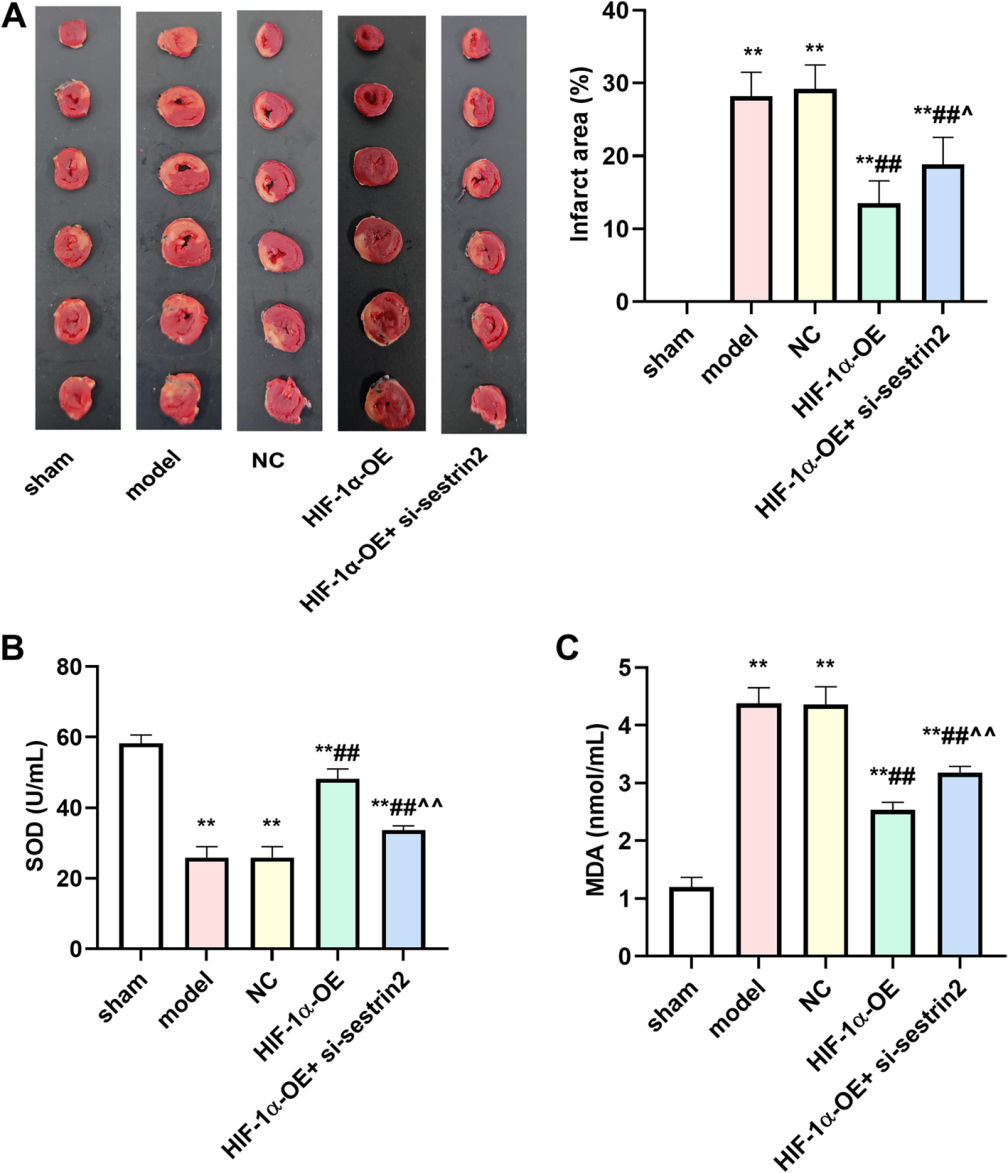
HIF-1α overexpression improved myocardial infarction area and oxidative stress state. **A** TTC staining was used to assess the myocardial infarction in different groups; **B** The level of SOD was assessed by corresponding SOD kit; **C** The level of MDA was assessed by corresponding MDA kit. *n* = 6. Compared to the control group, ^**^P < 0.01; Compared to model or NC group, ^##^*P* < 0.01; Compared to HIF-1α-OE group, ^^^*P* < 0.05, ^^^^*P* < 0.01

Figure 4A depicts the TTC data, revealing that the infarcted area in the model group exhibited a significant increase when compared to the sham group (*P* < 0.01). The HIF-1α-OE group showed a significant decrease in the infarcted area compared to the model and NC groups (*P* < 0.01). Nonetheless, the size of the infarcted region was greater in the HIF-1α-OE + si-sestrin2 group compared to the HIF-1α-OE group (*P* < 0.01). The content of SOD and MDA were detected. According to the data presented in Fig. 4B and C, the levels of SOD were reduced in the model and NC groups, whereas the levels of MDA were elevated in both groups. Notably, the level of SOD was elevated whereas MDA was reduced in the HIF-1α-OE group. In the HIF-1α-OE + si-sestrin2 group, there was a reduction in the content of SOD while an increase was observed in the content of MDA, in compared to the HIF-1α-OE group (*P* < 0.01).

### HIF-1α overexpression resulted in a positive impact on fibrosis of the myocardial tissue

**Fig. 5:**
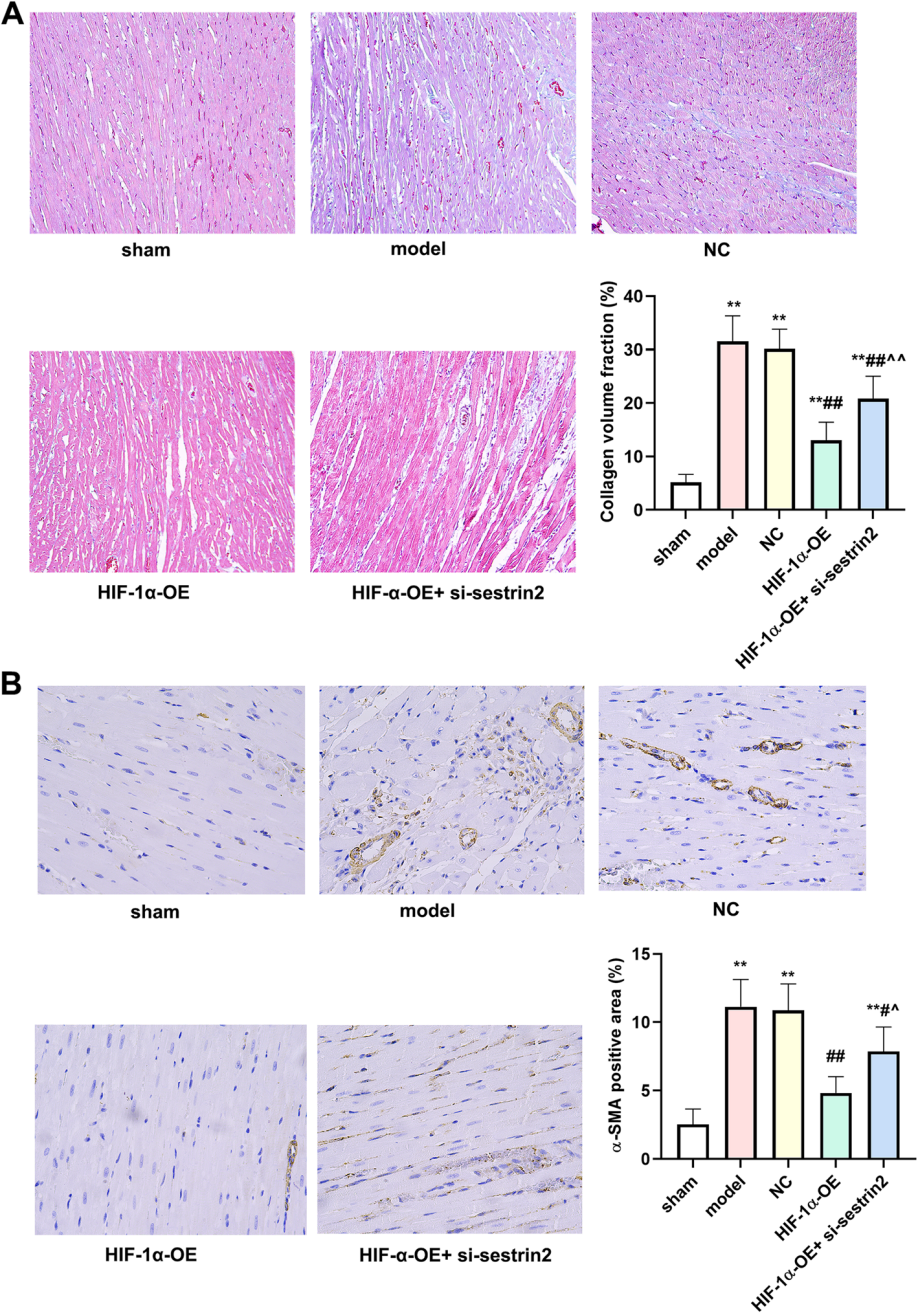
HIF-1α overexpression improved myocardial tissue fibrosis. **A** Masson staining was used to detect the collagen deposition (Scale bar: 100 μm); **B** IHC staining was utilized to investigate the expression of α-SMA (Scale bar: 100 μm). *n* = 6. Compared to the control group, ^**^P < 0.01; Compared to model or NC group, ^##^*P* < 0.01; Compared to HIF-1α-OE group, ^^^*P* < 0.05, ^^^^*P* < 0.01

Masson staining showed that there was no increase of collagen fibers in myocardial tissue of rats in the sham group. The myocardial tissue of the model group rats showed significant collagen fiber proliferation and deposition. The HIF-1α-OE group significantly reduced collagen deposition in myocardial tissue, and the protective effect of HIF overexpression on myocardial tissue in AMI rats. However, collagen deposition was significantly increased in the HIF-1α-OE + si-sestrin2 group compared to the HIF-1α-OE group. The results indicate that overexpression of HIF-1α can inhibit the deposition of collagen fibers in myocardial tissue of AMI rats (Fig. 5A). The expression level of α-SMA protein was detected by IHC staining. As presented in Fig. 4B, the expression level of α-SMA protein increased compared to the sham group (*P* < 0.01). Compared with model and NC groups, the expression level of α-SMA protein reduced in HIF-1α-OE group. In addition, the expression level of α-SMA protein enhanced in the HIF-1α-OE + si-sestrin2 group compared to the HIF-1α-OE group (Fig. 5B, *P* < 0.01).

### Overexpression of HIF-1α reduced cellular apoptosis in cardiac tissue

**Fig. 6:**
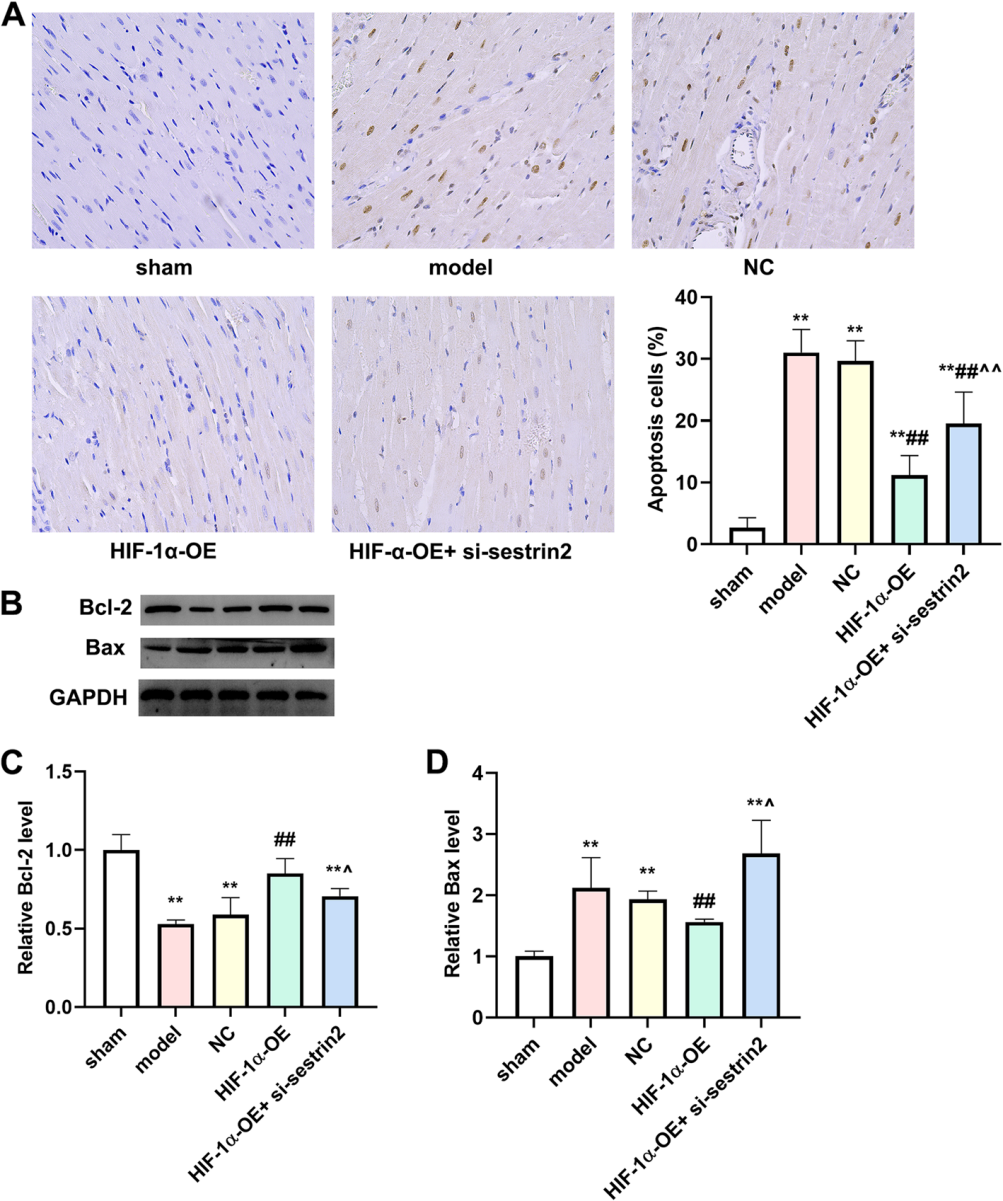
HIF-1α overexpression attenuated cell apoptosis in myocardial tissue. **A** TUNEL was used to assess the cells apoptosis in myocardial tissue (Scale bar: 100 μm, *n* = 6); **B** Western blotting was utilized to detect the expression levels of Bcl-2 and Bax in different groups (*n* = 3). The column diagram shows the quantification of Bcl-2 (**C**) and Bax (**D**). Compared to the control group, ^**^P < 0.01; Compared to model or NC group, ^##^*P* < 0.01; Compared to HIF-1α-OE group, ^^^*P* < 0.05, ^^^^*P* < 0.01

TUNEL data were illustrated in Fig. 6A, there was no apoptosis cells in myocardial tissue in the sham group. Compared to the sham group, apoptosis cells in myocardial tissue were increased in model and NC group. In the HIF-1α-OE group, the percentage apoptosis cells decreased compared to model and NC group. The percentage apoptosis cells increased in the HIF-1α-OE + si-sestrin2 group compared to the HIF-1α-OE group (*P* < 0.01). We then detected the expression levels of Bcl-2 and Bax. As expected, the expression level of Bcl-2 decreased while Bax increased in model and NC group compared with the sham group (Fig. 6B-D, *P* < 0.01). The expression level of Bcl-2 increased while Bax decreased in the HIF-1α-OE group compared with model and NC groups (*P* < 0.01). Nevertheless, the expression level of Bcl-2 decreased while Bax increased in the HIF-1α-OE + si-sestrin2 group compared with the HIF-1α-OE group (*P* < 0.01).

### The levels of protein expression were increased due to the overexpression of HIF-1α

**Fig. 7:**
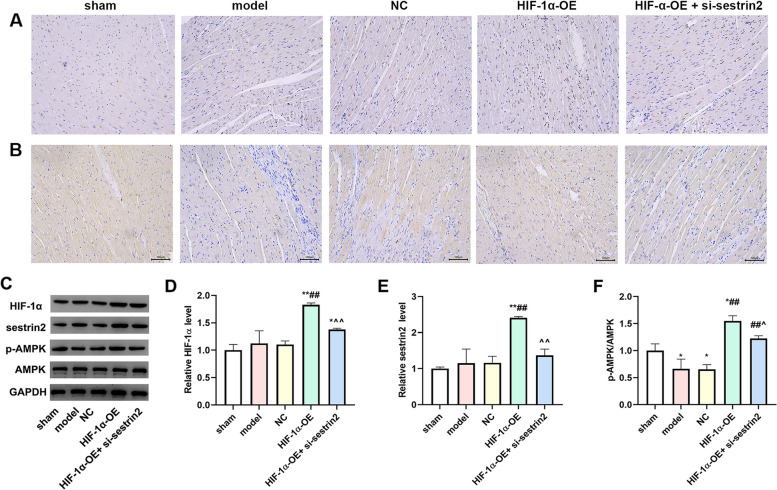
HIF-1α overexpression attenuated cell apoptosis in myocardial tissue. IHC staining was utilized to investigate the expression of HIF-1α (**A**) and sestrin2 (**B**) (Scale bar: 100 μm, *n* = 6); **C** Western blotting was utilized to detect the expression levels of HIF-1α, sestrin2, p-AMPK, and AMPK in different groups (*n* = 3). The column diagram shows the quantification of HIF-1α (**D**), sestrin2 (**E**) and p-AMPK/AMPK (**F**). Compared to the control group, ^*^P < 0.05, ^**^P < 0.01; Compared to model or NC group, ^#^*P* < 0.05, ^##^*P* < 0.01; Compared to HIF-1α-OE group, ^^^*P* < 0.05, ^^^^*P* < 0.01

IHC was utilized to investigate the expression of HIF-1α and sestrin2. As presented in Fig. 7A and B, the expression levels of HIF-1α and sestrin2 decreased in model and NC groups compared to the sham group. In the HIF-1α-OE group, the expression levels of HIF-1α and sestrin2 increased compared to model and NC groups. Nevertheless, the expression levels of HIF-1α and sestrin2 decreased in the HIF-1α-OE + si-sestrin2 group compared with the HIF-1α-OE group (*P* < 0.01). We further detected the proteins expression levels by western blotting. As expected, the changes of HIF-1α and sestrin2 were consistent with IHC results. In addition, the expression levels of p-AMPK and AMPK were detected. The expression level of p-AMPK decreased in model and NC group compared to the sham group. In the HIF-1α-OE group, the expression level of p-AMPK increased compared to model and NC group. However, the expression level of p-AMPK decreased in the HIF-1α-OE + si-sestrin2 group compared with the HIF-1α-OE group (Fig. 7C-F, *P* < 0.01).

## Discussion

AMI is a serious coronary heart disease [18]. AMI leads to ventricular remodeling, resulting in extensive fibrosis of myocardial cells, which ultimately leads to cardiac dysfunction and patient mortality [19]. Numerous studies confirmed that HIF-1α up-regulation ameliorated AMI injury [20, 21]. The initial focus of this research was to examine the damaged region using TTC. As TTC data presented, the infarcted area was the highest at day 14, and the lowest at day 28 after AMI, but the infarcted area was still approximately 25.8%. After AMI, we subsequently observed variations in the expression level of HIF-1α at various time intervals. At day 1 and 7, we observed that the HIF-1α expression level was significantly elevated. Following day 14, there was a reduction in the level of HIF-1α expression, displaying a subsequent downward pattern. The expression level of sestrin2 exhibited a comparable pattern to the expression of HIF-1α. From the current results, we thought that the HIF-1α could not sustain high expression. A previous study found that the HIF-1α expression level was increased to facilitate the protection of the myocardium [22]. Increasing HIF-1α expression could promoted angiogenesis [23]. Furthermore, HIF-1α overexpression in the heart during AMI is known to attenuate cardiac dysfunction [24]. The protective effect of HIF-1α in AMI was evident based on these findings. Nevertheless, there is a lack of definitive research on the variations in HIF-1α manifestation across various time intervals. We thought that in the early stage of AMI, the expression level of HIF-1α increased to prevent further exacerbation of myocardial tissue injury. Over time, the expression of HIF-1α was insufficient to support the repair of myocardial tissue injury. This hypothesis could explain our above experimental results. Therefore, we chose the day 28 as the following experiment time point.

Following various interventions, it was observed that the overexpression of HIF-1a led to a reduction in both LVEDD and LVESD, accompanied by an improvement in cardiac function through increased LVEF and LVFS. CK-MB and LDH play a crucial role in indicating damage to the heart muscle, subsequently leading to the alteration of the ventricular structure [25]. Following AMI, the levels of LDH and CK-MB were elevated in the model and NC groups. However, the overexpression of HIF-1a resulted in a reduction in LDH and CK-MB levels. Nevertheless, the administration of si-sestrin2 partially counteracted the inhibitory impact of HIF-1a overexpression. H&E staining results showed that the overexpression of HIF-1a enhanced the morphological alterations in the myocardial tissue and reduced the inflammatory reaction. IL-6 and TNF- α iso-inflammatory cytokines are key factors in initiating cascade inflammatory responses [26]. Cell releases IL-6 and TNF- α can induce the decline of myocardial cell vitality and apoptosis, accelerate the secretion of a large amount of collagen and myocardial fibrosis in myocardial tissue, damage myocardial function. We found that HIF-1a overexpression declined the contents of IL-6 and TNF- α, while si-sestrin2 treatment partial increased the contents of IL-6 and TNF- α. Wang et al. found that the higher stability in HIF-1α- extracellular vesicles induced the stronger cardiac function in the rats [14]. The overexpression of HIF-1α in extracellular vesicles may improve the morphological alterations and inflammatory response. Our results were in line with their investigation.

There will be oxidative stress reaction after AMI [27]. In the present study, HIF-1α overexpression inhibited the content of MDA, enhanced the level of SOD, diminishedinfarction area, collagen deposition, and expression of α-SMA protein. Nevertheless, the co-administration of si-sestrin2 led to an increase in inflammatory factors, myocardial injury markers, collagen deposition, and α-SMA expression, suggesting that the protective effect of HIF-1α overexpression was reversed by inhibiting sestrin2. The protective effect of sestrin2 on individuals with anxiety and depression myocardial infarction has been reported, and this effect was linked to AMPK [28]. According to our research, the sestrin2/AMPK signaling was found to be associated with the protective impact of HIF-1αoverexpression. Nevertheless, we solely confirmed the impact of elevated HIF-1α and sestrin2 through animal trials. In the future, extensive clinical studies are required to validate the precise signaling cascade linking the two, in order to identify more efficient therapeutic targets for the clinical management of AMI. Seo et al. reportes that sestrin2 increases HIF-1α degradation via AMPK–PHD regulation that contributes to inhibition of tumorigenesis in vitro and in vivo [29]. Hu et al. suggests that HIF-1α expression is induced under hypoxia, which mediates AMPK activation and mTOR inhibition, thus promoting autophagy and tumor cell survival [30]. However, the effect of HIF-1α expression was controversial. In the current study, there were still limitations. First, we only detected the infarction area, and HIF-1α expression level up to day 28, therefore, a longer period of time more than day 28 maybe contribute to deep understanding the effect of HIF-1α. Furthermore, we only investigate the expression of HIF-1α, sestrin2, and AMPK, there are maybe other effective factors. In the future study, a long time period need to be investigated, and a large amount of clinical research is needed to confirm the specific signaling cascade between the HIF-1α and sestrin2.

To summarize, HIF-1a overexpression enhanced the cardiac function in rats, decreased the myocardial infarction area, improved myocardial injury, and diminished the rate of apoptosis in myocardial cells. The findings suggested that the HIF-1a overexpression decrease the cardiac damage in rats following AMI, and this effect is linked to the sestrins2/AMPK signaling pathway. Our research provide a novel sight on the management of AMI through the overexpression of HIF-1a.

## Data Availability

The data analyzed during this study are available from the corresponding author on reasonable request.
